# The prevalence and socio-demographic correlates of hypertension among women (15–49 years) in Lesotho: a descriptive analysis

**DOI:** 10.1186/s12889-022-12960-0

**Published:** 2022-03-22

**Authors:** Mapitso Lebuso, Nicole De Wet- Billings

**Affiliations:** Demography and Population Studies, School of Social Sciences, Faculty of Humanities, University of the Witwatersrand, Johannesburg, 2000 South Africa

**Keywords:** Hypertension, High blood pressure, Socio-demographic factors

## Abstract

**Background:**

Hypertensive disorders are among the leading conditions for severe maternal morbidity across all regions and have a major impact on health care costs. This study aimed to identify the prevalence and its associated socio-demographic correlates of hypertension among women of the reproductive ages in Lesotho.

**Methods:**

The study used the Lesotho Demographic and Health Survey (2014 LDHS) data set. A total of 3353 women of childbearing age (15–49 years) whose blood pressure was measured were used for analysis. The blood pressure readings were categorized according to the JNC7 cut-offs. The dependent variable of this study is hypertension. Both bivariate and binary logistic regressions were performed to determine socio-demographic correlates of hypertension.

**Results:**

Results from this study revealed that one out of every five respondents of the study had hypertension compared to 23% who were in the prehypertension stage. The situation adds to the overall future risk of hypertension. About 30% percent who were at the hypertension stage were either living with a partner or widowed. The odds of being hypertensive were significantly 9.78 times higher among women aged 45–49 years [CI: 6.38–15.00]. Other factors associated with hypertension among women of the reproductive ages were “living with a partner” [OR 3.55:95% CI: 1.76–7.16], widowed [OR 2.61:95% CI: 1.89–3.60], and residing in the Maseru district [OR 2.12: 95% CI: 1.49–3.03].

**Conclusion:**

Chances of being diagnosed with high blood pressure increased with an increase with the age of the respondents. Age was found to be the most definite positive significant socio-demographic correlate of hypertension among women in Lesotho. To control hypertension, primary prevention strategies should target the identified high-risk -older age groups, the ever-married as well as prehypertensive women.

## Background

Africa is expected to have 216.8 million hypertensive people by 2030. Over 54.6 million cases of hypertension were estimated in 1990, 92.3 million cases in 2000 and 130.2 million cases in 2010 respectively. Hypertension is prevalent in Africa [1, 2]. Similarly, hypertension is widespread in Sub-Saharan Africa, its consequences include among others cardiovascular diseases and increased risk in morbidity and mortality [3]. According to the latest WHO data published in 2018, the WHO STEPS of 2012 and other surveys done in 2001 and 2012 show that the prevalence of hypertension in the population was 31%. Hypertension related deaths in Lesotho reached 536 or 1.91% of the total deaths [Who.int/ncds/surveillance/steps/Lesotho_2012_STEPS_fact_sheet.pdf]. Hypertension is the 9th leading cause of death in the world, and Lesotho is ranked number 4 at 46.81per 100 000 according to the world rankings.

Hypertension is a major cause of morbidity among adult patients in Lesotho; it is among the five causes of female admission into hospitals. Hypertension is also the third most common cause of outpatient attendance and one of the leading causes of admission to public health [4–7]. The high prevalence of hypertension exerts a tremendous public health crisis [8, 9]. Mashea et al. [8], discovered that obstetric haemorrhage and hypertensive disorder escalates mortality by 31.4% and 28% respectively. The objective of this study is to identify prevalence and its associated socio-demographic correlates of hypertension among women aged 15–49 years in Lesotho. Prevalence of hypertension remains high (one in 3 persons are hypertensive) and it remains a challenge in the country despite concerted efforts made by the Lesotho government and development partners to curb it. Previous studies conducted in the country made investigations on hypertension treatment and control in primary care setting as well as knowledge of disease and medications among hypertension patients. The child-bearing women’s demographic and social factors which could be predictors of hypertension have not been examined in Lesotho. The study is intended to fill that gap in the literature.

## Methods

This is a secondary data analysis of cross-sectional data of the 2014 Lesotho Demographic and Health Survey (LDHS). These are women of childbearing age (15–49 years) who had ever given birth in the five years preceding the 2014 LDHS. The total unweighted female population in the LDHS was 6,621. In determining the variable of interest, respondents were asked whether they were ever diagnosed with high blood pressure by a doctor or a nurse [10]. Blood pressure readings were taken from 3353 who were included in the final analysis. About fifteen percent (705) respondents were ever diagnosed with high blood pressure. The individual female dataset for the 2014 LDHS was used for this study and the data were extracted and processed using Stata version 14.

### The outcome variable

In this study, hypertension is the outcome variable, which was defined using the WHO classification and categorized using the JNC7 cut-offs. The categorization was done with the use of blood pressure records of women taken from the 2014 Lesotho Demographic and Health Survey [11].

This variable is derived from the survey question of “Ever been diagnosed with high blood pressure by a doctor or a nurse?”. If the response is “yes”, then the inclusion criteria which was used was for those whose hypertension levels were 140 + mmHg (systolic) or 90 + mmHg (diastolic) or above. The outcome variable was categorized as hypertension stage 1, that is, those with SBP ≥ 140 (mmHg) or DBP of ≥ 90 (mmHg), then Hypertension stage 2, as those with SBP ≥ 160 (mmHg) or DBP SBP ≥ 100 (mmHg) [12].

### Independent variable

The independent variables of the study were socio-demographic characteristics such as age, marital status, place of residence, region/district, religion, level of education and occupation.

### Statistical analyses

Cross-tabulations, bivariate and logistic regression analyses were done. At the bivariate level, the percentage distribution of the study sample was presented by the selected socio-demographic characteristics of the women. The correlation was tested using the Pearson correlation coefficient. Binary logistic regression was used to determine socio-demographic correlates of hypertension among women aged 15–49 years in Lesotho. A *p*-value of < 0.05 was considered statistically significant. All analyses were carried out using version 14 of the STATA software.

### Ethical consideration

The Lesotho DHS can be downloaded from the website and is free to use by researchers for further analysis. In order to access the data from DHS MEASURE, a written request was submitted to the DHS MACRO, and permission was granted to use the data for this survey.

## Results

### Socio-demographic characteristics of the respondents

Table 1 depicts the socio-demographic characteristics of respondents. Regarding the profile of women, 23.29% were aged 15–19 years while 7.40% were aged 45–49 years. More than three quarters (67%) were rural dwellers and 14% were residing in the Maseru district compared to 8% from Quthing and Qacha’s Nek districts respectively. About one percent (0.80%) were living with their partners compared to 54% who were married. More than half (51%) of the women had completed the secondary level of education (Table 1). Thirty eight percent were members of the Roman Catholic Church compared to 0.02% of the Hindu religion. About 17% of the women’s occupation was sales while only 1.3% reported being agricultural employees.

**Table 1:** The socio-demographic characteristics of women in Lesotho, 2014

Characteristics	N(6621)	N(705) Ever diagnosed with high blood pressure
Age groups	n(%)	n%)
15–19	1242 (23.29)	29 (4.11)
20–24	1300 (19.63)	93 (13.19)
25–29	1072 (16.19)	94 (13.33)
30–34	907 (13.70)	116 (16.45)
35–39	728 (11.00)	118 (16.74)
40–44	582 (8.79)	114 (16.17)
45–49	490 (7.40)	141 (20.00)
Place of residence		
Urban	2202 (33.26)	254 (36.03)
Rural	4419 (66.74)	451 (63.97)
Region/district		
Botha-bothe	593 (8.96)	47 (6.67)
Leribe	785 (11.86)	93 (13.19)
Berea	760 (11.48)	77 (10.92)
Maseru	930 (14.05)	147 (20.85)
Mafeteng	624 (9.42)	96 (13.62)
Mohale's hoek	621 (9.38)	66 (9.36)
Quthing	556 (8.40)	42 (5.96)
Qacha's-nek	558 (8.43)	56 (7.94)
Mokhotlong	605 (9.14)	43 (6.10)
Thaba tseka	589 (8.90)	38 (5.39)
Marital Status		
Single	2201 (33.24)	98 (13.90)
Married	3556 (53.71)	470 (66.67)
Living with partner	53 (0.80)	12 (1.70)
Widowed	471 (7.11)	83 (11.77)
Divorced	96 (1.45)	13 (1.84)
No longer living together/separated	244 (3.69)	29 (4.11)
Level of education		
No education	81 (1.22)	10 (1.42)
Primary	2665 (40.25)	290 (41.13)
Secondary	3354 (50.66)	327 (46.38)
Higher	521 (7.87)	78 (11.06)
Religion		
Roman catholic church	2514 (37.97)	281 (39.86)
Lesotho evangelical church	1133 (17.11)	135 (19.15)
Methodist	111 (1.68)	10 (1.42)
Anglican church	453 (6.84)	58 (8.23)
Seventh day Adventist	40 (0.60)	6 (0.85)
Pentecostal	1682 (25.40)	138 (19.25)
Other Christian	540 (8.16)	64 (9.08)
Islam	11 (0.17)	2 (0.28)
Hindu	1 (0.02)	0 (0.00)
No religion	65 (0.98)	4 (0.57)
Other	71 (1.07)	7 (0.99)
Professional/technical/managerial	295 (10.84)	49 (12.89)
Clerical	132 (4.85)	23 (6.05)
Sales	475 (17.46)	69 (18.16)
Agricultural—self-employed	270 (9.92)	35 (9.21)
Agricultural—employee	35 (1.29)	3 (0.79)
Household and domestic	420 (15.44)	40 (10.53)
Services	314 (11.54)	54 (14.21)
Skilled manual	266 (9.78)	42 (11.05)
Unskilled manual	353 (12.97)	43 (11.32)
Don't know	161 (5.92)	22 (5.79)
		*N* = 380

### Percentage of respondents diagnosed with high blood pressure

As expected, women (63.97%) who resided in the rural areas were more likely to be hypertensive than their urban counterparts (36.03%). Majority were 45–49 years old, and were from Maseru district (20.85%). They had secondary education, belonged to the Roman Catholic Church and their occupation was more likely to be in the sales sector.

**Fig. 1:**
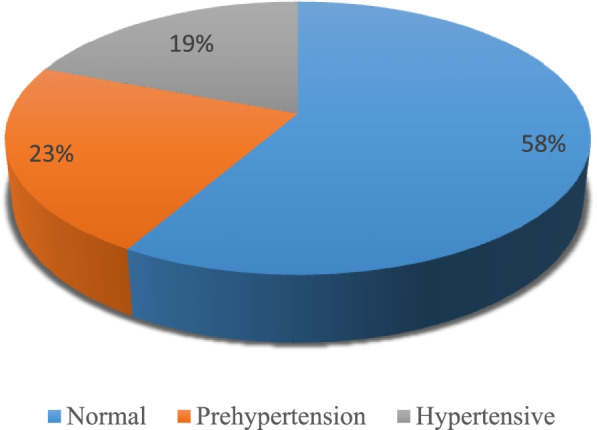
Percentage distribution of women by hypertension status, Lesotho, 2014

Figure 1 displays information on the hypertension status of women. The hypertension status has been divided in *normal (those with “systolic BP (SBP)* <  = *120–129(mmHg) and/or diastolic* <  = *80- 84 (mmHg))*, prehypertension (systolic BP (SBP) 130 -139*(mmHg)* and/or diastolic 85–89(mm Hg) and hypertensive (those with SBP ≥ 140 (mmHg) and/or DBP of ≥ 90 (mmHg))

Based on Fig. 1, 19% of women have hypertension, compared to 23% and 58% who have prehypertension and normal blood pressure.

Table 2 presents chi-square results of hypertension status by socio-demographic characteristics of women in Lesotho.

**Table 2:** Hypertension status by socio-demographic factors among women aged 15–49 years in Lesotho, 2014

Characteristics	**Normal Blood pressure** (systolic < = 120–129 and diastolic < = 80- 84)	**Prehypertension** (systolic 130–139 and/or diastolic 85–89)	**Hypertension** **(systolic** ≥ 140–159 and/or diastolic ≥ 90–99)	Total	Pearson’s Chi square
Age group					
15–19	556 (70.83)	161 (20.51)	68 (8.66)	785	< 0.000
20–24	421 (68.01)	125 (20.19)	73 (11.79)	619	
25–29	335 (62.92)	122 (22.93)	75 (14.10)	532	
30–34	239 (52.07)	128 (27.89)	92 (20.04)	459	
35–39	168 (46.80)	86 (23.96)	105 (29.29)	359	
40–44	129 (44.64)	52 (17.99)	108 (37.31)	289	
45–49	72 (28.92)	67 (26.91)	110 (44.18)	249	
Place of residence					
Urban	647 (59.80)	231 (21.35)	204 (18.85)	1082	0.439
Rural	1273 (57.60)	510 (23.08)	427 (19.32)	2210	
**Region/district**					
Botha-bothe	144 (27.30)	80 (27.30)	69 (23.55)	293	< 0.001
Leribe	241 (61.95)	91 (23.39)	57 (14.65)	389	
Berea	233 (61.97)	74 (19.68)	69 (18.35)	376	
Maseru	265 (58.50)	89 (19.65)	99 (21.85)	453	
Mafeteng	167 (55.12)	67 (22.11)	69 (22.77)	303	
Mohale's hoek	194 (60.82)	72 (22.57)	53 (16.66)	319	
Quthing	137 (50.00)	68 (24.82)	69 (25.18)	274	
Qacha's-nek	165 (60.00)	56 (20.36)	54 (19.68)	275	
Mokhotlong	191 (61.22)	73 (23.40)	43 (15.38)	312	
Thaba tseka	183 (61.41)	71 (23.83)	44 (14.77)	298	
**Marital Status**					
Single	724 (66.85)	231 (21.33)	128 (11.82)	1083	< 0.000
Married	953 (54.21)	417 (23.72)	388 (22.07)	1758	
Living with partner	18 (54.55)	5 (15.15)	10 (30.30)	33	
Widowed	133 (52.57)	46 (18.18)	74 (29.25)	253	
Divorced	28 (57.14)	11 (22.45)	10 (20.41)	49	
No longer living together/separated	64 (55.17)	31 (26.72)	21 (18.10)	116	
**Level of education**					
No education	23 (56.10)	9 (21.95)	9 (21.95)	41	0.030
Primary	748 (55.86)	313 (23.38)	278 (20.86)	1339	
Secondary	1012 (61.00)	364 (21.94)	283 (17.06)	1659	
Higher	137 (54.15)	55 (21.74)	61 (24.11)	253	
**Religion**					
Roman catholic church	717 (56.15)	297 (23.26)	263 (20.60)	1277	< 0.001
Lesotho evangelical church	331 (57.57)	126 (21.91)	118 (20.52)	575	
Methodist	29 (49.15)	15 (25.42)	15 (25.42)	59	
Anglican church	99 (49.01)	53 (26.42)	50 (24.75)	202	
Seventh day Adventist	17 (80.95)	2 (9.52)	2 (9.52)	21	
Pentecostal	528 (62.93)	184 (21.93)	127 (15.14)	839	
Other Christian	157 (62.30)	52 (20.63)	43 (17.06)	252	
Islam	0 (0.00)	1 (33.33)	2 (66.67)	3	
No religion	16 (51.61)	7 (22.58)	8 (25.81)	31	
Other	26 (78.79)	4 (12.12)	3 (9.09)	33	
**Occupation**					
Professional/technical/managerial	73 (50.34)	29 (20.00)	43 (31.88)	148	0.017
Clerical	34 (49.28)	13 (18.84)	22 (25.96)	69	
Sales	111 (47.23)	61 (25.96)	63 (26.81)	235	
Agricultural—self-employed	75 (51.02)	38 (25.85)	34 (23.13)	147	
Agricultural—employee	13 (72.22)	2 (11.11)	3 (16.67)	18	
Household and domestic	144 (62.61)	51 (22.17)	35 (15.22)	230	
Services	85 (54.84)	41 (26.45)	29 (18.71)	155	
Skilled manual	62 (45.93)	42 (31.11)	31 (22.96)	135	
Unskilled manual	90 (52.63)	46 (26.90)	35 (20.47)	171	
Don't know	47 (58.02)	14 (17.28)	20 (24.69)	81	
**Total**	**1920 (58.32)**	**741 (22.51)**	**323 (19.17)**	**3353 (100)**	

*p* < 0.05 is considered statistically significant (Chi-Square test)

A total of 58.32%, 22.51%, and 19.17% of the females had normal blood pressure, were prehypertensive, and had hypertension respectively The bivariate analysis show that age, region, marital status, level of education, religion and occupation have a significant association with hypertensive status of women. The findings revealed that 44% of females aged 45–49 were found to be more hypertensive compared to other age cohorts.

Females in the professional/technical/managerial occupation (32%) and with high level of education (24%) had higher levels of blood pressure readings of SBP ≥ 140 (mmHg) or DBP of ≥ 90 (mmHg). Furthermore, 25% and 24% of females from the Quthing and Botha-Bothe districts were hypertensive while 30% and 29% of women who were either living with a partner or widowed had a blood pressure reading of SBP ≥ 140 (mmHg) or DBP SBP ≥ 90 (mmHg). While on the other hand, 25% of women who belong to Methodist and Anglican Church were reported to be at prehypertensive stage.

Table 3 displays binary logistic regressions of the Odds Ratio (OR) of hypertension status and socio-demographic factors among women aged 15–49 years. Age has been found to have a positive influence on hypertension. Thus, compared with women aged 15– 19 years, the odds of being hypertensive were significantly higher among females aged 25–29 [OR: 2.06; CI: 1.2 3,2.91], 30–34[OR: 3.23; CI: 2.11,4.93], 35–39[OR: 4.47; CI: 2.92,6.85], 40–44 [OR: 5.69; CI: 3.71,8.75] and 45–49 [OR: 9.78; CI: 6.38,14.99], respectively.

**Table 3:** Odd Ratios for socio-demographic factors associated with prevalence of hypertension among women aged 15–49 years who had at least one live birth in the 5 years preceding the survey in Lesotho, 2014

Socio-demographic characteristics	Odds, 95% C.I	*P* value
**Age**		
15–19	RC	
20–24	1.89 (1.22,2.91)	0.004*
25–29	2.06 (1.34, 3.17)	0.001*
30–34	3.23 (2.11,4.93)	< 0.000*
35–39	4.47 (2.92, 6.85)	< 0.000*
40–44	5.69 (3.71, 8.75)	< 0.000*
45–49	9.78 (6.38, 15.00)	< 0.000*
**Level of education**		
No education	RC	
Primary	0.73 (0.39, 1.47)	0.376
Secondary	0.68 (0.33, 1.37)	0.276
Higher	0.81 (0.39, 1.70)	0.582
**Place of residence**		
Urban	RC	
Rural	0.92 (0.78, 1.09)	0.316
**Marital Status**		
Single	RC	
Married	1.71 (1.39, 2.16)	< 0.000*
Living with partner	3.55 (1.76, 7.16)	< 0.000*
Widowed	2.61 (1.90, 3.60)	< 0.000*
Divorced	1.60 (0.86, 3.00)	0.140
Separated	1.54 (0.99, 2.41)	0.057
**Region/district**		
Botha-bothe	RC	
Leribe	1.39 (0.95, 2.02)	0.088
Berea	1.23 (0.84, 1.82)	0.289
Maseru	2.12 (1.49, 3.03)	< 0.000*
Mafeteng	2.05 (1.40, 2.99)	< 0.000*
Mohale's hoek	1.26 (0.84, 1.88)	0.262
Quthing	0.98 (0.63, 1.52)	0.924
Qacha's-nek	1.33 (0.87, 2.01)	0.185
Mokhotlong	0.91 (0.58, 1.41)	0.663
Thaba tseka	0.79 (0.51, 1.25)	0.320
Religion		
Roman catholic church	RC	
Lesotho evangelical church	1.06 (0.85, 1.33)	0.598
Methodist	1.02 (0.51, 2.02)	0.966
Anglican church	1.16 (0.85, 1.58)	0.353
Seventh day Adventist	1.19 (0.49, 2.91)	0.697
Pentecostal	0.73 (0.57, 0.91)	0.005*
Other Christian	1.04 (0.77, 1.39)	0.818
Islam	1.15 (0.25, 5.35)	0.858
Hindu	-	-
No religion	0.42 (0.15, 1.18)	0.100
Other	0.74 (0.33, 1.65)	0.461
**Occupation**		
Professional/technical/managerial	RC	
Clerical	1.14 (0.66, 1.99)	0.640
Sales	0.97 (0.64, 1.45)	0.867
Agricultural—self-employed	0.88 (0.55, 1.42)	0.612
Agricultural—employee	0.66 (0.19, 2.29)	0.508
Household and domestic	0.68 (0.43, 1.07)	0.093
Services	1.15 (0.75, 1.77)	0.524
Skilled manual	1.05 (0.66, 1.66)	0.840
Unskilled manual	0.77 (0.49, 1.21)	0.253
Don't know	0.91 (0.52, 1.58)	0.728

*Significant *p*-values: *p* < 0.005; 95% Confidence intervals (CI); *OR* odds ratio, *RC* Reference Category

Married women were found to be 1.71 times more likely to be hypertensive than single women. Likewise, widows were 2.61 times more likely to be hypertensive than single women counterparts. This was true as well for couples living together [OR: 3.55; CI: 1.76,7.16] (Table 3).

Conversely, women who belonged to Pentecostal church were found to be 0.73 times less likely to be hypertensive compared to their Roman Catholic Church fellows [CI: 0.59,0.91].

## Discussion

The objective of the study was to determine prevalence of hypertension and also identify socio-demographic correlates of hypertension in women in Lesotho. Baseline analysis has illustrated that a high proportion of women were aged 15–19 years, most (67%) of them resided in rural areas. More than half (54%) were married while 51% of the women had completed the secondary level of education.

In agreement with similar studies conducted in other African countries like Ethiopia, the study demonstrated that older age groups are a strong factor associated with hypertension. The proportion of women who were diagnosed with high blood pressure increases with an increase in age, the odds of being hypertensive were significantly higher among women aged 45–49 years. This is in line with other studies where the risks of hypertension increase with age [1, 13]. Thinyane, 2015 also discovered that age was among factors associated with poor blood pressure in Lesotho.

Moreover, there were higher odds for the ever-married (married, divorced, and widowed) to be diagnosed with hypertension. In Ghana, Tuoyire (2018) found significantly higher odds of hypertension for married, cohabiting, and previously married adults. It seems that married and widowed/divorced/separated, women were at higher risk of having hypertension and this could be due to the inevitable “vicissitudes of marriage.” [14]. Likewise, Wickham, 2001 found out that marital stress significantly increases the likelihood of earlier hypertension among long term married women. Using secondary data made it impossible to evaluate other confounding factors, such as stress levels among women, especially those living with a partner or widowed.

Specifically, the study addresses hypertension, a topic of particular relevance to Lesotho given its high rate of maternal mortality. Literature suggests a link between hypertension and maternal morbidity and mortality. However, there is a limitation to the generalizability of the results because only female data was utilized.

## Conclusion

This study showed that age was associated with hypertension among a sample of the women adult population in Lesotho. The study further suggests that 741 (22.10%) of the respondents were in prehypertension stage, which adds to the overall future risk of hypertension. The socio-demographic correlates of hypertension among women include: advancement in age, living with partner, being married, being widowed, and living in Maseru and Mafeteng districts. While the primary prevention strategies should start with Basotho women in high-risk groups, the importance of focusing on prehypertensive individuals should not be overlooked because it indicates a future risk of hypertension.

## Data Availability

The data that support the findings of this study are available at measuredhs.com.

## Abbreviations

DBP: Diastolic Blood Pressure
DHS: Demographic and Health Survey
LDHS: Lesotho Demographic and Health Survey
MOH: Ministry of Health
SBP: Systolic Blood Pressure
USAID: United States Agency for International Development
WHO: World Health Organization
